# Effect of acid–base balance on postoperative course in children with hypoplastic left heart syndrome after the modified Norwood procedure

**DOI:** 10.1097/MD.0000000000007739

**Published:** 2017-08-25

**Authors:** Marcin Gładki, Tomasz Składzień, Rafał Żurek, Elżbieta Broniatowska, Elżbieta Wójcik, Janusz H. Skalski

**Affiliations:** a Department of Pediatric Cardiac Surgery, University Children's Hospital, Jagiellonian University; b Anaesthesiology and Intensive Care Clinic, University Hospital; c Department of Bioinformatic and Telemedicine, Jagiellonian University, Krakow, Poland.

**Keywords:** acid–base balance, hypoplastic left heart syndrome, Norwood procedure

## Abstract

Hypoplastic left heart syndrome (HLHS) is a congenital heart defect that requires 3-stage cardiac surgical treatment and multidirectional specialist care. The condition of newborns in the first postoperative days following the modified Norwood procedure is characterized by considerable hemodynamic instability that may result in a sudden cardiac arrest. It is believed that the most important cause of hemodynamic instability is the fluctuations in redistribution between pulmonary and systemic blood flow.

The paper analyzes the postoperative course in 40 neonates with HLHS following the modified Norwood procedure performed under deep hypothermic cardiopulmonary bypass hospitalized in Cardiac Surgical Intensive Care Unit (CSICU) in the years 2014–2015. For all hospitalized children, the arterial blood acid–base balance (ABB) parameters (pH, pO_2_, pCO_2_, HCO_3,_ base excess (BE), and lactic acid) were measured 2 times a day during the first 5 postoperative days. The main goal of the studies is to analysis of ABB parameters and their influence on the clinical state of newborns with HLHS. Several descriptors were concerned to describe the neonates clinical state: the date of the surgery (the day of life when the child was operated on), the duration (number of days) of mechanical ventilation employment, the time of hospitalization in intensive care unit, and the total duration of treatment in CSICU.

The statistical analysis of the particular ABB parameters revealed a significant dependence (*P* < .001) between the values of pH, pO_2_, pCO_2_, HCO_3_, BE, lactic acid, and all concerned descriptors of the newborn clinical state.

The article shows that monitoring the ABB parameters, proper interpretation of the results, and appropriate modification of pharmacotherapy and respiratory treatment are crucial for therapeutic results and survival rates in neonates with HLHS after the modified Norwood procedure.

## 1 Introduction

Statistically about 1.25% children with congenital heart defects are patients with single ventricle heart.^[1,2]^ Hypoplastic left heart syndrome (HLHS) is a complex heart defect which is associated with a high risk of death at each treatment stage. Numerous centers worldwide collaborate to ensure the safest perinatal conditions for children with HLHS, provide proper and prompt diagnostic management, and determine optimal performing of consecutive stages of surgery and effective postoperative treatment. Multi-center interdisciplinary collaboration offers hope for developing optimal diagnostic and therapeutic management in children with HLHS. At the first stage of HLHS treatment, various procedures are employed, depending on experience of a given center and anatomy of the defect. This may be a hybrid procedure, the classic Norwood operation or a heart transplant. Nevertheless, in the majority of centers, the modified Norwood procedure is performed.^[3–5]^

Despite progress in diagnostic management of heart defects, including also the prenatal period, as well as in preoperative treatment, modification of surgical techniques, strategy of cardiopulmonary bypass and postoperative management, the mortality rates among children with HLHS continue to be high. It is estimated that approximately 20% children (i.e., every fifth patient) subjected to the Norwood procedure do not survive this stage of treatment.^[6–9]^

Newborns after the modified Norwood procedure manifest considerable hemodynamic instability in the first postoperative days, which may result in sudden death. The principal cause of postoperative hemodynamic instability is blood flow disturbances between pulmonary and systemic circulation. Changes in vascular resistance in pulmonary circulation in the first hours and days of life may lead to increased blood flow through the pulmonary vascular bed and resultant abnormalities of systemic circulation, organ hypotension, and hypoperfusion.^[10,11]^

Particularly dangerous for the patient's life are coronary circulation disturbances which result in arrhythmias and even cardiac arrest. The main importance in postoperative management of children with HLHS is maintaining appropriate acid–base balance in order to preserve the proper ratio of pulmonary (Qp) and systemic blood flow (Qs). Appropriate parameters of mechanical ventilation and selection of proper vasodilating medications allow for regulating vascular resistance, both systemic and pulmonary, and thus for maintaining an appropriate ratio of pulmonary to systemic blood flow (Qp/Qs).

The main purpose of the paper is the analysis of acid–base balance parameters and their effect on the clinical state of newborns with HLHS following the modified Norwood procedure. The assessment of acid–base balance parameters before surgery and their effect on postoperative state of children are concerned as additional goal of the studies.

## 2 Material and methods

The analysis focuses on the clinical course in 40 neonates with hypoplastic heart syndrome following the modified Norwood procedure performed under deep hypothermic cardiopulmonary bypass at the Department of Pediatric Cardiac Surgery, Polish–American Children's Hospital, Jagiellonian University, Krakow, in the years 2014–2015. The retrospective analysis included the results of acid–base balance (ABB) parameters (pH, pCO_2_, pO_2_, HCO_3_, base excess (BE), and lactic acid) in arterial blood in the first 5 postoperative days measured routinely 2 times a day in all patients hospitalized in Cardiac Surgical Intensive Care Unit (CSICU) at Department of Pediatric Cardiac Surgery. All the children were operated on by the Norwood procedure as modified by Sano, under deep hypothermic cardiopulmonary bypass. For the 3 (7.5%) of 40 operated patients, respiratory support employing extracorporeal membrane oxygenation (ECMO) was necessary in the postoperative period. These patients were excluded from the statistical analysis. Table 1 contains statistical description of pregnancy and perinatal period of the neonates included in the further analysis.

**Table 1:**
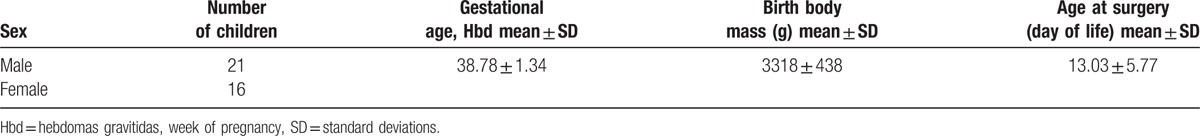
Characterization of children with hypoplastic left heart syndrome subjected to the analysis.

The statistical analysis was performed using the STATISTICA version 10 software. The Spearman's rank correlation test and Pearson's correlation test were applied to determine the relationships between continuous variables. In view of the large deviations from normality, Friedman's test was performed to compare the parameters that were regularly (10 times) measured postoperatively. Statistical significance was assumed at *P***≤** .05. The study was retrospective and based on test results originating from medical records. No approval by the Ethics Committee was necessary.

## 3 Results

**Figure 1:**
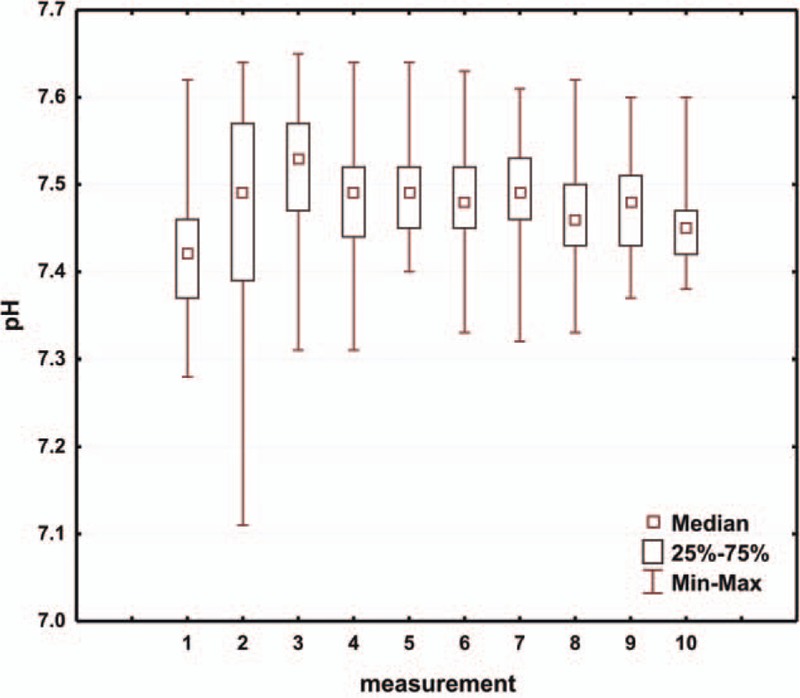
Box and whisker plot for values of pH.

**Figure 2:**
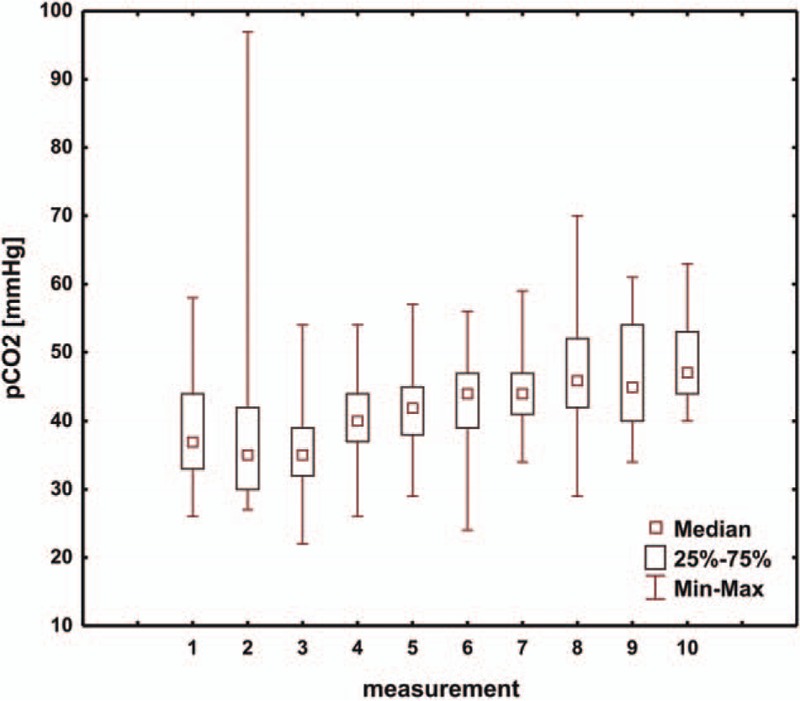
Box and whisker plot for values of pCO_2_.

**Figure 3:**
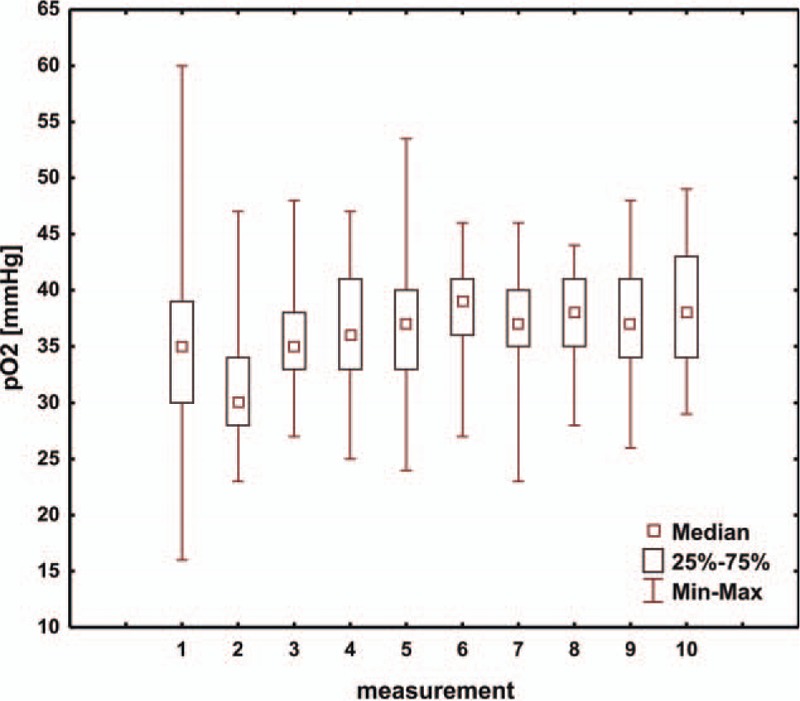
Box and whisker plot for values of pO_2_.

**Figure 4:**
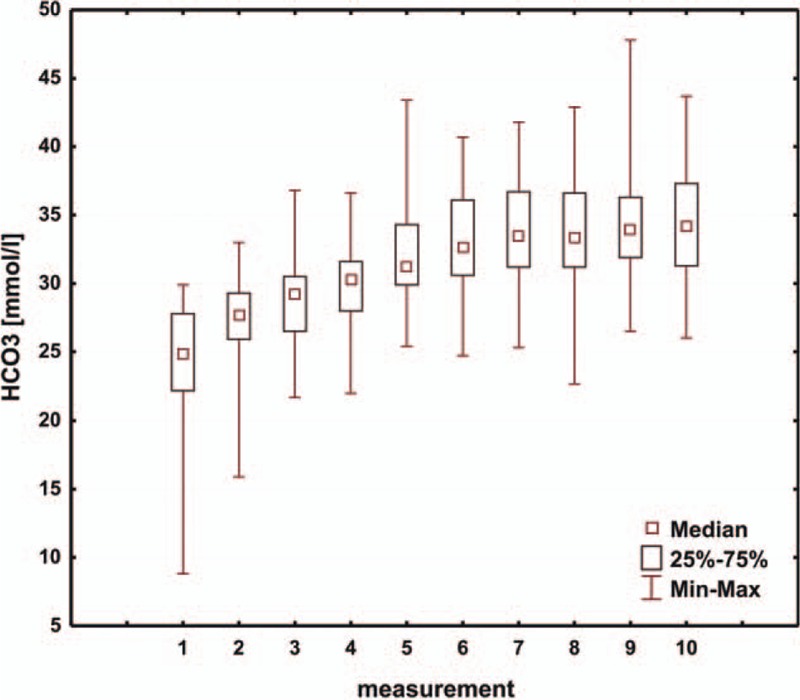
Box and whisker plot for values of HCO_3_.

**Figure 5:**
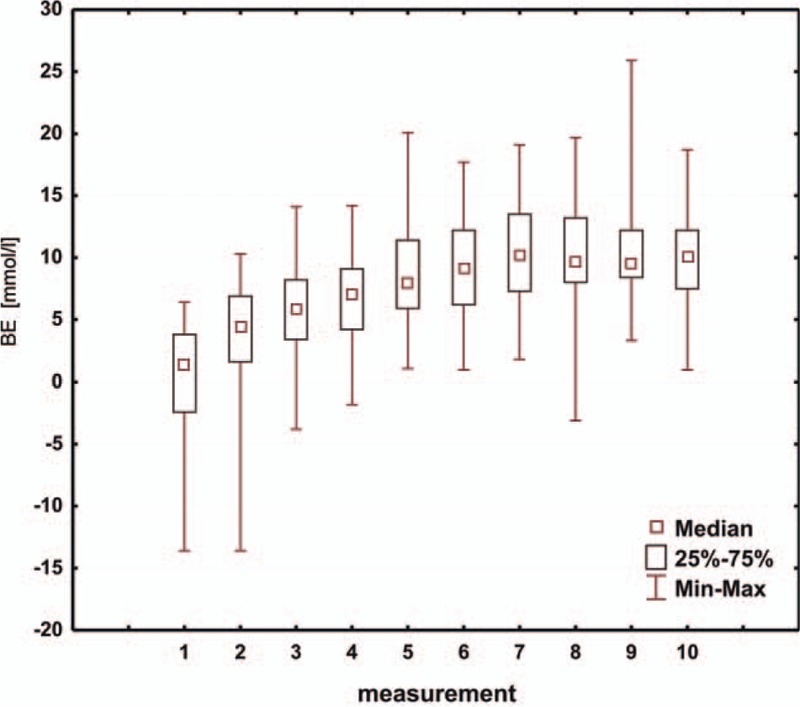
Box and whisker plot for values of BE. BE = base excess.

**Figure 6:**
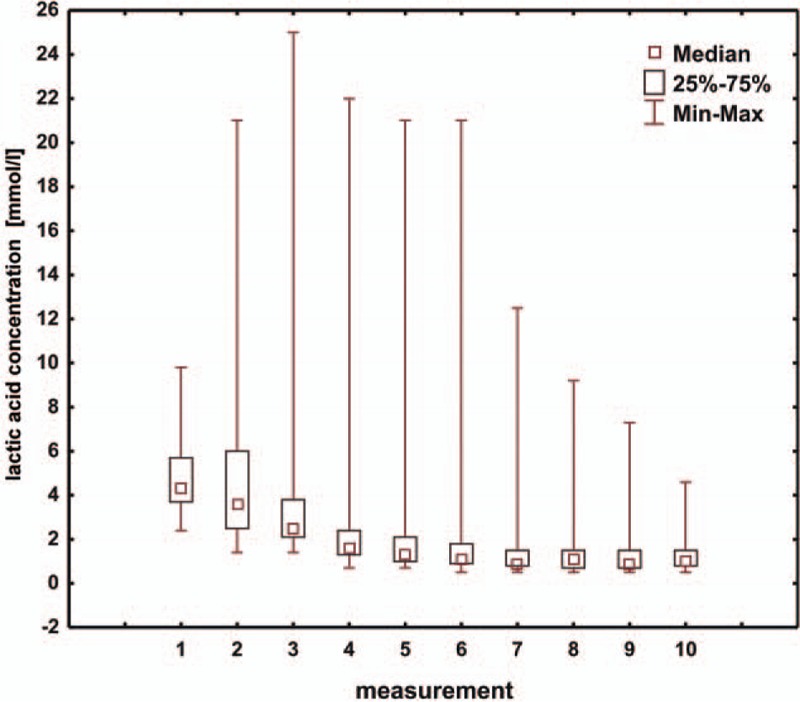
Box and whisker plot for values of lactic acid levels.

The analysis included the results of arterial blood gas measurements from the first 5 days of treating the child in the intensive care unit following the modified Norwood procedure. The first value of arterial blood gas test was defined as the result obtained upon the child arriving in the intensive care unit. Subsequent tests were performed at 7.00 PM on the same day and at 7.00 AM and 7.00 PM of each subsequent day. The mean pH, pCO_2_, pO_2_, HCO_3_, and BE values in consecutive days of hospitalization in the intensive care unit are presented in Table 2 and are shown in Figs. 1–5. Table 3 illustrates the mean values of lactic acid concentration in consecutive tests: the results are shown in Fig. 6.

**Table 2:**
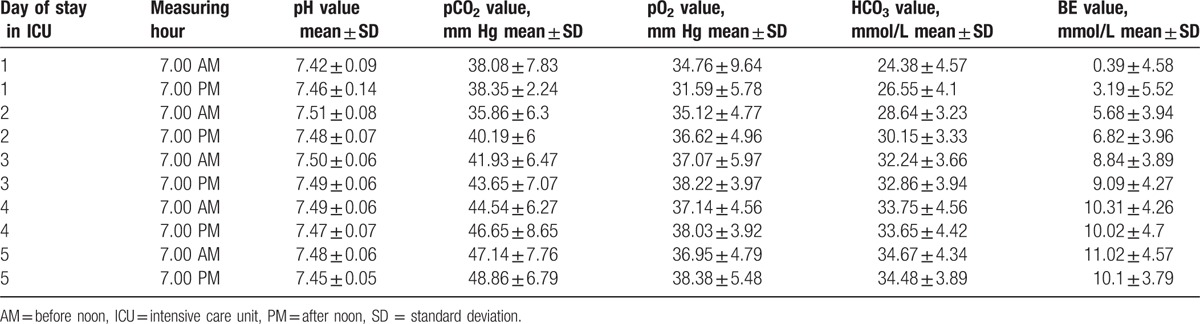
Mean values and standard deviations of pH, pCO_2_, pO_2_, HCO_3,_ and BE in consecutive days of hospitalization in the intensive care unit.

**Table 3:**
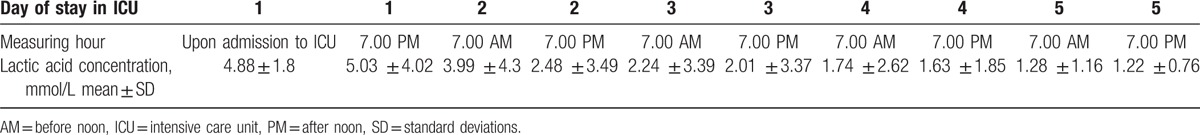
Mean values and standard deviations of arterial blood lactic acid concentration determined over consecutive 5 days.

The mean duration of mechanical ventilation in the analyzed group was 19.38 (± 16.96) days. The mean of the total hospitalization time was 53.22 (± 22.18) days. The mean time of hospitalization in the intensive care unit was 35.95 (± 19.82) days. The mortality rate in the analyzed group was 5.4% (2 children).

A statistically significant negative correlation was demonstrated between the birth body mass and duration of mechanical ventilation in the postoperative period (Spearman's ρ = –0.43, *P* = .007) and the birth body mass and duration of hospitalization in the intensive care unit (Spearman's ρ = –0.40, *P* = .013), whereas the correlation between the birth body mass and total hospitalization time turned out not significant (*P* = .079).

No statistically significant correlation was demonstrated between the age (day of life) when the child was operated on and the time (number of days) of employing mechanical ventilation (*P* = .93), duration of hospitalization in the intensive care unit (*P* = .93), and the total duration of hospitalization (*P* = .63).

The Friedman test was used for determination which of the clinical state descriptors (the day of life when the child was operated on, duration (number of days) of mechanical ventilation employment, time of hospitalization in the intensive care unit, and total duration of hospitalization) influence on acid–base balance parameters. Due to the applied Friedman test, all these clinical condition descriptors were categorized into 4 levels defined by the first quartile, median, and the third quartile. It was proved that all measured ABB parameters are statistically dependent on all the studied clinical state descriptors: the day of life when the child was operated on, duration (number of days) of mechanical ventilation employment, time of hospitalization in the intensive care unit, and total duration of hospitalization (all *P*-values are below .001).

Moreover, a significant dependence was shown between lactic acid concentration on days 1, 2, 3, 4, and 5 after the surgery and age (day of life) when the child was operated on, duration of mechanical ventilation, number of days of hospitalization in the intensive care unit, and total hospitalization time (*P* < .001 for all the Friedman tests).

## 4 Discussion

According to the current literature, the principal risk factors affecting the survival of patients after the Norwood procedure are the following: the anatomical variant of hypoplastic left heart syndrome, necessity of anticonvulsant medication administration upon discharge from hospital, gestational age (duration of pregnancy), feeding via a gastrointestinal tube on the day of discharge, and the duration of the vasoactive medications administration in the postoperative period.^[12]^ However, there is relatively lack of reports including the analysis of ABB values in the initial postoperative days that would address the aspect of assessing postoperative risk of the Norwood procedure.

In patients with congenital heart defects manifesting as single ventricle heart, with mutual mixing of the systemic and pulmonary circulation, just as in the case of children after the Norwood procedure, it is of the utmost importance to appropriately select the ventilation parameters to ensure the balanced Qp/Qs blood flow. In the case of inappropriate ventilation of the patient, excessive pulmonary blood flow may develop, what will cause hemodynamic instability.^[10,11]^

Due to the disturbances of blood flow balance, pulmonary blood flow and blood oxygenation increase at the cost of decreased systemic flow, including coronary blood flow, what causes heart ischemia. Some authors suggest enriching the mixture of respiratory gases with carbon dioxide,^[13,14]^ which exerts a potent vasoconstrictive effect on pulmonary vessels with a minimal effect on the resistance of peripheral vessels. No carbon dioxide was employed in the mixture of respiratory gases in our patients.

The most important aspect of effective treatment offered in intensive care units is maintaining homeostasis in the patient. To calculate the appropriate balance between pulmonary flow (Qp) and systemic flow (Os), a mathematical equation developed by Barne was used. The proper Qp/Qs ratio equals 1.0.^[15]^

The importance of the appropriate pulmonary to the systemic flow ratio in children after the Norwood procedure is emphasized by Rossi et al,^[11]^ who analyzed in his report the ABB results in 13 patients following the Norwood procedure. Two children died 24 hours postoperatively. In his study, Rossi calculated the value of Qp/Qs based on the value of venous saturation that equaled 96%. The value of Qp/Qs in 6 of 13 children immediately after surgery was 2.0.

In their investigations, Strauss et al^[16]^ analyzed the values of the first postoperative ABB and did not demonstrate significant differences between the groups of surviving children as compared to patients who died in the postoperative period. When we compare the ABB values, we note that in the study of Strauss et al^[16]^, the last determination was performed immediately before completing the procedure, whereas in our clinical material, it was done immediately after completing the operation. It may be thus assumed that the values are comparable. The children discussed by Strauss et al^[16]^ showed lower values of pH, pCO_2_, and BE and higher values of pO_2_ as compared to the respective values in our material. We strove to maintain pO_2_ not exceeding 40 mm Hg. Maintaining the value of pO_2_ on a moderate level aims at controlling pulmonary resistance and in consequence, at maintaining stable, balanced pulmonary and systemic blood flow. Such a management method allows for achieving hemodynamic stability in a child after the Norwood procedure.

The ABB values in the first postoperative days affected the total time of mechanical ventilatory support. Consecutive values of pH (Fig. 1), starting from the third measurement, that is, approximately 24 hours after surgery, confirm the stabilization of ABB parameters. Similarly, when we analyze the values of pCO_2_ in consecutive measurements (Fig. 2), we note parameter stabilization starting from the third measurement, that is, after approximately 24 hours following the surgical procedure. Our attention is drawn to a tendency toward a slow increase in the pCO_2_ value in consecutive postoperative days.

Moreover, when we look at the values of pO_2_ (Fig. 3), we observe stabilization after the third measurement, that is, approximately 24 hours after the operation.

While analyzing the HCO_3_ values (Fig. 4) in consecutive measurements, we note that the interval for all the measurements is similar and that, starting from the first measurement, the HCO_3_ values show an increasing trend. Stabilization of the HCO_3_ values occurs starting from the fourth measurement, that is, 36 hours after the procedure. Observing consecutive BE values (Fig. 5), we see that the interval for all the measurements is similar and that, starting from the first measurement, the BE values show an increasing tendency. Stabilization of the BE values is seen starting from the fourth measurement, or approximately 36 hours after the operation. Additionally, the preoperative management and optimal preparation of the child for the surgery are also important. In order to perform a more detailed and accurate analysis of patients with HLHS after the modified Norwood procedure, further studies in this group of children are necessary.

One of the risk factors that increase mortality rates after the Norwood procedure, pointed out by investigators is the age of the child on the surgery day which is above 7 days of life.^[17]^ In our studies, the mean age on the day of operation was 13 days. The day when the procedure was performed affected the ABB values, and thus the condition of the child and the prognosis.

Another prognostic factor is the lactic acid level.^[18]^ In the discussed group of patients, we noted a gradual decrease in lactic acid levels (Fig. 6). Stabilization of the lactic acid concentration values starts from the fourth measurement, that is, approximately at 36 hours postoperatively. The postoperative level of lactic acid affected the duration of hospitalization in the intensive care unit, total hospitalization time, and duration of mechanical ventilation.

## 5 Conclusions

In neonates with hypoplastic left heart syndrome (HLHS) after the modified Norwood procedures, monitoring the parameters of acid–base balance, proper interpretation of the results, and appropriate modification of pharmacotherapy and respiratory treatment have fundamental importance for therapeutic results and survival rates.
